# Maintaining fixation by children in a virtual reality version of pupil perimetry

**DOI:** 10.16910/jemr.15.3.2

**Published:** 2022-09-19

**Authors:** Brendan L. Portengen, Marnix Naber, Demi Jansen, Carlijn van den Boomen, Saskia M. Imhof, Giorgio L. Porro

**Affiliations:** Department of Ophthalmology, University Medical Center Utrecht, The Netherlands; Experimental Psychology, Utrecht University, The Netherlands

**Keywords:** Eye movement, eye tracking, saccades, virtual reality, pupillometry, attention

## Abstract

The assessment of the visual field in young children continues to be a challenge. Children often do not sit still, fail to fixate stimuli for longer durations, and have limited verbal capacity to report visibility. Therefore, we introduced a head-mounted VR display with gazecontingent flicker pupil perimetry (VRgcFPP). We presented large flickering patches at different eccentricities and angles in the periphery to evoke pupillary oscillations, and three fixation stimulus conditions to determine best practices for optimal fixation and pupil response quality. A total of twenty children (3-11y) passively fixated a dot, counted the repeated appearance of an animated character (counting task), and watched an animated movie in separate trials of 80s each (20 patch locations, 4s per location). The results showed that gaze precision and accuracy did not differ significantly across the fixation conditions but pupil amplitudes were strongest for the dot and count task. The VR set-up appears to be an ideal apparatus for children to allow free range of movement, an engaging visual task, and reliable eye measurements. We recommend the use of the fixation counting task for pupil perimetry because children enjoyed it the most and it achieved strongest pupil responses.

## Introduction

To this day visual field assessment in children remains challenging due to certain characteristics of standard automated perimetry (SAP; e.g., Humphrey Field Analyzer, Octopus perimeter). These include the task’s subjectiveness, the requirement of fixation on a target, uncontrollable learning effects, and the need for prolonged attention. Due to these disadvantages, perimetry tests performed with young children and patients that suffer from cortical damage tend to produce unreliable results (19; 25; 37).

Pupil perimetry was developed as an objective alternative to SAP, using the pupillary response to light stimuli across the visual field as a measure of visual sensitivity (9; 34; 41). Conventional pupil perimetry set-ups consist of a monitor and a stand-alone eye tracker. Pupil perimetry has not yet been performed in children even though it circumvents most of the aforementioned challenges in evaluating the visual field with SAP (i.e., subjectiveness and the need for fixation on a target). The reason why pupil perimetry has not yet been applied in children may stem from the remaining requirement to stay seated while fixed in a forehead-chinrest.

Here we propose a novel implementation of gaze-contingent flicker pupil perimetry (23) through the use of a head-mounted device (HMD) with virtual reality (VR) technology (VRgcFPP). VR applications in the ophthalmologic practice are relatively new, but promising (1; 6; 8; 17; 30; 35, 36). Particularly, VR allows for freedom of head movement, a child-friendly and engaging environment, and eye measurements using a built-in eye tracker. Eye trackers used for pupil perimetry mostly consist of sophisticated and expensive solutions, such as the Eyelink 1000 Plus (SR Research, Ontario, Canada) or the Tobii Pro Spectrum (Tobii, Danderyd, Sweden), but recent developments now allow high-quality eye-tracking with a HMD.

Also, the immersive environment that VR provides introduces new possibilities to engage children during assessments. Increased attention has been shown to evoke stronger pupillary responses to stimuli (5; 15; 22) which in turn increases discriminative power (29). Here we questioned how it can be ensured that children show sustained attention for the visual stimuli in a VR environment. An instruction to keep attention will not suffice for young and/or neurologically impaired children. To maintain fixation, a simple fixation point will not be interesting enough to look at, but a fixation object of a type that is too distracting might lead to unwanted pupillary reactions (i.e., noise in the pupil response data). All these aspects could thus hypothetically lead to decreased quality of measurements, denoting its importance to find a balance between increased attention towards fixation and maintaining a good signal-to-noise ratio in the pupillary measurements.

### Aims

In summary, the aim of this study is to explore whether visual field examination using a virtual reality version of pupil perimetry (VRgcFPP) provides strong pupil responses in children, and what fixation task is best suited for them and what fixation task provides the most reliable results.

## Methods

### Participants

The participants consisted of 20 healthy children aged 3 to 11 years old (mean age and SD 7.2 ± 2.4, 14 male). The sample size was similar to prior studies in the field (1; 10; 24; 29). All children had normal or corrected-to-normal visual acuity and no history of visual or neurological disorders. Participants were not tested for visual acuity, but parents were questioned about any signs of visual problems to ensure that vision of the child was good (for details, see procedure). The experiment was approved by the local ethical committee of Utrecht University (approval number FETC19-006) and conformed to the ethical considerations of the Declaration of Helsinki. Participants gave written informed consent together with their caretakers prior to participation. Both participants and caretakers were clearly instructed of their right to withdraw consent and informed that the experiment could be halted prematurely. Researchers observed the child during the experiment for any sign of reluctance or distress, after which the experiment would immediately be ended. Lastly, they received (financial) reimbursement (€8,- per hour) and a phone-based VR headset for participation.

### Apparatus

The tests were conducted either in the laboratory or at the residence of the participants. A BTO 17W1090 laptop (BTO, IJsselstein, The Netherlands) with Windows 10 operating system (Microsoft, Redmond, Washington) was used to run the test. The VR environment was built with Unity software (version 2019.4; Unity Technologies, San Francisco, CA, USA). Connected to the laptop was an HTC (HTC Corporation, Taoyuan, Taiwan) Vive Pro Eye VR headset. It consisted of dual 3.5-inch OLED screens with a resolution of 1440x1600 pixels per screen and a refresh rate of 90 Hz to display stimuli. Pupil diameter and gaze were recorded with the built-in Tobii eye tracker (Tobii, Danderyd, Sweden; 90 Hz sampling rate, 0.5-1.1-degree accuracy of gaze angle) and the VIVE SRanipal Runtime and SDK. Adjustment of the HMD and eye tracker calibration (5-point grid) took ~1 min. Two base stations at opposite positions located real-time head position with SteamVR Tracking 2.0. Stimulus properties (i.e., fixation target, frequency, location, size, and order) were inputted with Python software (version 3.7; xml.etree.cElementTree and numpy packages; Python Software Foundation, https://www.python.org/).

### Fixation target conditions

**Figure 1.**
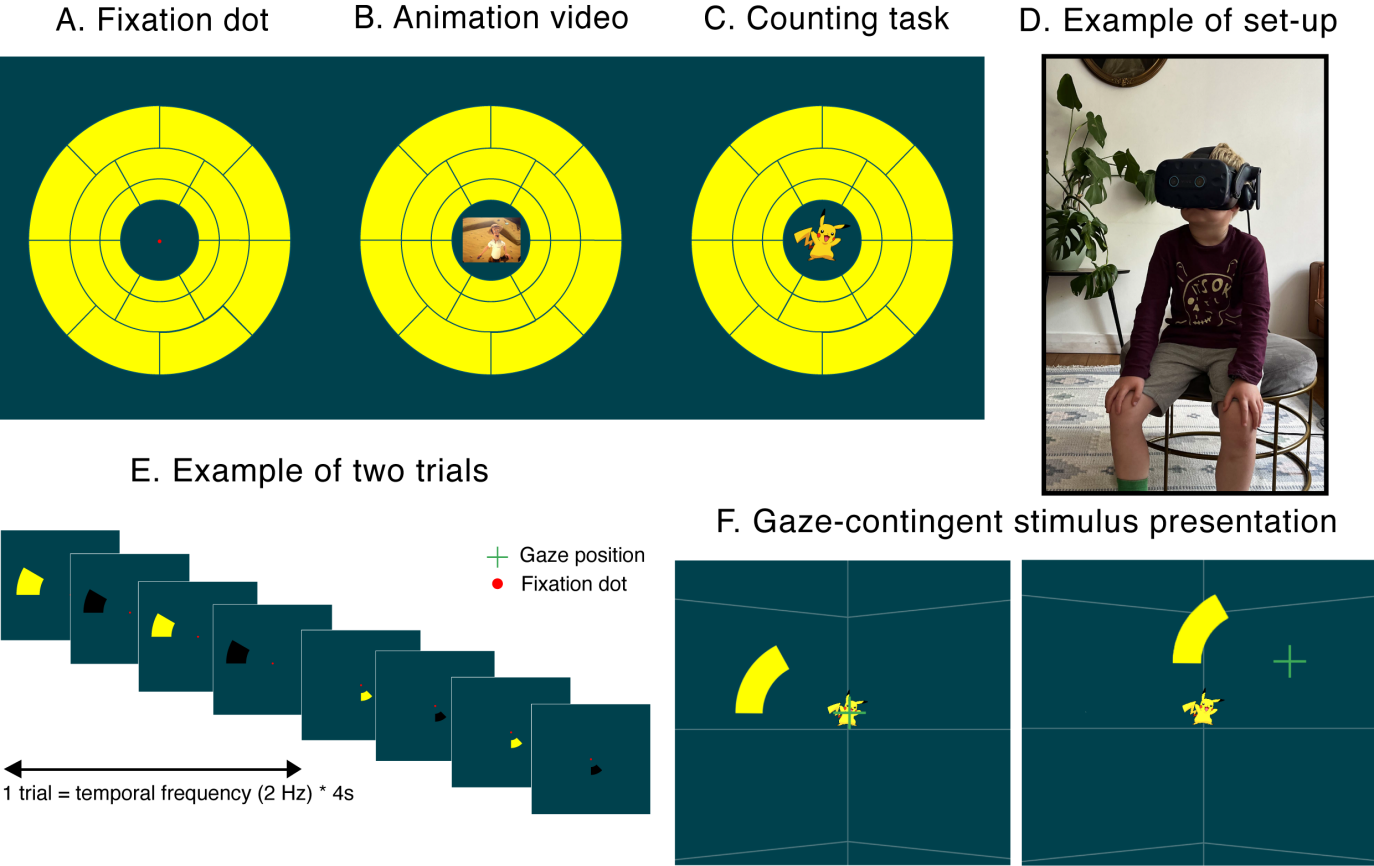
The three fixation target conditions used in this study; a red fixation point (A), an animation video (B), and a counting task (C). Children were seated in a chair where the headset was positioned. A picture of a participant at home (6 years old) is shown in (D). All fixation targets were displayed at a fixed position in the middle of a dark blue virtual reality environment. The 2 Hz flickering yellow-and black stimuli consecutively appeared at the 20 stimulus locations (E). To ensure accurate retinotopic stimulation stimuli were presented in a gaze-contingent manner (F), i.e., online correction of stimulus locations for saccades from fixation target. Note that thin white lines were added to the background to create a sense of depth in the virtual reality environment. Note that the green gaze position cross was not shown during the experiment but is here shown to illustrate the gaze-contingent presentation paradigm.

The three fixation target conditions used in this study consisted of the presentation of (i) a simple red fixation dot, similar to fixation targets used in standard automated perimetry and conventional pupil perimetry (Figure 1A), (ii) an animated child-friendly video of an archeologist in Egypt (chosen for its relatively low luminance, color and spatial contrast; adopted from https://youtu.be/j6PbonHsqW0) with muted sound (Figure 1B), and (iii) an engaging counting task in which participants were asked to count the appearances of an animated character at fixation (Pikachu; Pokémon, The Pokémon Company, Minato, Tokyo, Japan, see Figure 1C). This character appeared 14 times within the 80 second trial at varying intervals. All fixation targets were placed on a fixed location within the VR environment independent of head or gaze position. To prevent large saccades in reaction to the fixation target conditions, the three fixation targets were made small by placing them at a simulated distance of 16 m.

### Environment & stimuli

A dark blue (30% luminance for optimal luminance and color contrast; Portengen et al., submitted) background served as the VR environment. To reduce Simulator Sickness (due to sensory mismatch; Reason, 1978) a sense of depth was simulated through a virtual red platform upon which the participants were placed, and thin dome-like lines. Stimuli were superimposed on the background. The stimuli consisted of black‑yellow flickering wedges presented across 20 stimulus locations in randomized order within the inner 60 degrees field of vision and positioned around one of three fixation targets with a simulated distance of 10 m, see Figure 1. Note that the inner 16 degrees of the visual field were not stimulated to allow for the fixation conditions to be visible. The black-yellow wedges flickered for 4 seconds (i.e., to collect sufficient pupil data but also keep the experiment relatively short) at a 2 Hz rate and were superimposed on a complementary dark blue background (Figure 1E). The 2 Hz flicker frequency is the optimal balance between enough number of evoked pupil responses in a relatively short time window and strong enough pupil responses that can be picked up reliably by the eye tracker (Portengen et al., in prep). The gaze-contingent stimulus presentation (i.e., the eye tracking software follows the subject’s direction of gaze fixation and updates the position of the flickering stimuli real-time to reflect changes in direction of gaze; Figure 1F) ensured accurate retinotopic stimulation despite the presence of saccades (23).

### Procedure

After the informed consent procedure, children and their caretakers completed a demographic questionnaire to ensure no neurologic, ophthalmologic or attentional disorders were present. Upon completion participants were seated on a chair in the center of the room, where the VR HMD was fitted to the child’s head (Figure 1D). A short adjustment period (~1 min) followed after this. Here the child could look around the VR environment; young children were made aware of the red platform underneath them: “Stay seated, because the floor is lava!”. Aside from using this joke as a way to make the children feel more comfortable, the platform also created an extra sense of depth in the otherwise “empty” VR environment. After calibration with a 5-point calibration grid, the experiment started. This consisted of three blocks, each with a fixation target, a 5-second adjustment period and flickering stimuli at 20 locations across the visual field. The children were instructed to fixate their gaze at the fixation target in the middle of the environment. The younger children were encouraged to fixate the center of the screen by verbally expressing the following instructions; (i) during the fixation dot condition, the experimenters reminded a child to keep looking at the dot when its gaze strayed from it, (ii) for the animation video, the experimenters occasionally asked the participating child what was going on in the video, and lastly (iii) children were positively reinforced whenever they counted the appearance of a Pikachu during the counting task condition. The experiment lasted for 240 seconds (3 fixation target blocks * 20 stimulus locations * 4 second stimulus duration). The child could take a break between each block. Total experiment duration, including all trials, breaks, and (re)calibration was on average 15 minutes. Pupils were measured binocularly to estimate convergence and thus focus of depth in the VR environment. The dual OLED screens allowed a sense of depth in the VR environment to prevent VR induced Simulator Sickness.

### Analysis

First stimulus location onsets functioned as start events for the event-related analysis of the continuous pupil output of the integrated eye tracker. From the pupil data blink episodes were detected and removed using an automated detection blink method by looking for crossings of a speed threshold of 4 standard deviations (SD) above the mean. The removed blink epochs were interpolated with a cubic method. Next, pupil data were baseline-corrected to enhance inter-subject comparability. A high-pass Butterworth filter (3^rd^ order, 1 Hz cut-off frequency) and a low-pass filter (3^rd^ order, 10 Hz cut-off frequency) followed to remove slow pupil diameter changes and high‑frequency noise, respectively. Pupil traces per stimulus location were converted to power values in the frequency domain using a fast Fourier transform. The power at 2 Hz reflected the pupil oscillation amplitude and served as the main dependent variable. Furthermore, we were interested in how well each stimulus fixation paradigm retained a child’s attention. For this we calculated gaze distance from the fixation target. Distance means and SDs of saccades across fixation conditions were compared. One-way repeated measures ANOVA and paired double-sided t-tests (post-hoc tests) determined statistical significance of pupil amplitudes, fixation accuracy, and fixation precision between fixation conditions. All analyses were performed using Python software (version 3.7; Python Software Foundation, https://www.python.org/). The raincloud plot was created using software developed by (2).

## Results

**Figure 2.**
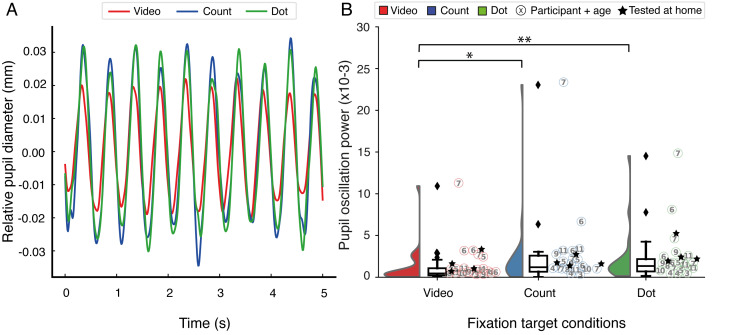
Relative pupil diameter over time for all subjects across fixation target conditions are shown in (A). Pupil traces are averaged across stimulus locations and participants. A raincloud plot depicting the average pupil oscillation powers per fixation target condition are plotted in (B) where the video fixation task (left) is red, the counting task (middle) is blue, and the fixation dot task (right) is green. Individual participants and their age (in years) and test location (i.e., lab or at home (n = 4)) are plotted across fixation conditions. The results show no distinct differences in patterns across age groups or test locations. The fixation dot and counting task conditions provided significantly larger pupil powers than the video fixation target (* = *p* < .05, ** = *p *< .01).

In our study, we set out to investigate whether visual field examination using a virtual reality version of pupil perimetry is feasible and which fixation target condition evoked strongest pupil responses. To do this, pupil data were analyzed to inspect adequate pupil responses to the 2 Hz stimulation. Figure 2A shows the 2 Hz oscillatory pattern of the pupil traces, averaged across all children, reflecting the stimulus on- and offsets (see Supplementary Figure S1 for separate plots with 95% confidence intervals).

Next, pupil oscillation powers were compared between fixation target conditions (one-way repeated measures ANOVA; *F*(2,38) = 3.87, *p* = .030, *partial η^2^* = 0.17). Post-hoc comparisons demonstrated stronger pupil powers of the fixation dot condition (*t*(19) = 3.05, *p* = .007) and the counting task condition (*t*(19) = 2.12, *p* = .047) when compared to the video fixation target. There existed, however, no statistical difference between fixation dot and counting task (*t*(19) = 0.73, *p* = .470). See Figure S2-4 in the Supplementary Materials for the average pupil traces, the average pupil oscillation powers and the pupil oscillation power spectra per stimulus location across fixation target conditions per participant.

**Figure 3.**
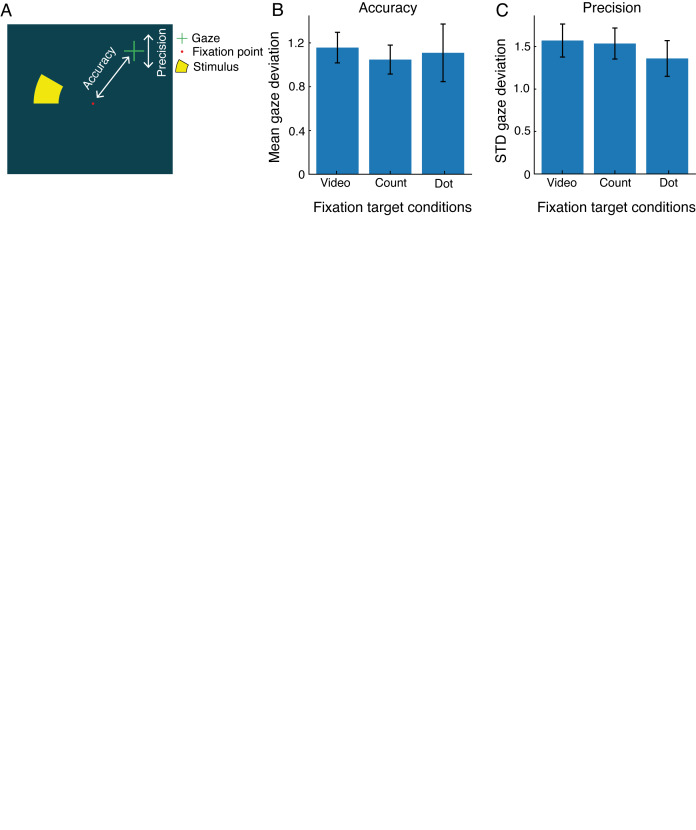
Gaze accuracy and precision of participants; accuracy is defined as the mean gaze deviation from fixation target and precision as the standard deviation of this gaze deviation (A). The gaze accuracy (B) and precision (C) did not significantly differ across fixation target conditions.

Furthermore, we explored which fixation target ensured best fixation behavior. The fixation error from fixation target center (i.e., gaze distance during fixation loss) provided an indication of interest and attention, see Figure 3. A one-way repeated measures ANOVA revealed that gaze accuracy (i.e., mean gaze deviation from fixation center; *F*(2,38) = 2.20, *p* = .120) and gaze precision (i.e., standard deviation of gaze deviation; *F*(2,38) = 1.18, *p* = .320) did not differ significantly across fixation target condition. These results imply that the fixation target conditions did not influence fixation error in the children studied.

After every experiment the investigators queried which fixation target conditions participants enjoyed the most. Almost all (18 out of 20 children) preferred the counting task. The two oldest participants (≥10 years old) favored the fixation dot, likely because the Pokémon character and video targeted matched best with the interest of children younger than 10.

## Discussion

In our study, we set out to investigate whether visual field examination using a virtual reality version of pupil perimetry (VRgcFPP), is feasible in children by testing whether strong pupil responses could be evoked. Moreover, the secondary objectives of this study were to investigate (i) what fixation task is best suited for children, and (ii) which fixation target best captured a child’s attention.

The fixation dot and counting task conditions provided strongest pupil responses. One possible explanation for the weaker pupil responses during the animated video fixation target task is the lack of covert attention for the flickering stimuli (15; 16; 21; 29). In addition to attention, the pupil also responds to luminance contrast (39), color hue (7; 12; 38; 40), and spatial frequency (3; 14; 39). The video’s higher luminance and spatial contrast in comparison to the other two fixation targets could have interfered with the luminance and color contrast between stimulus and background. Interestingly, one participant (S2; see Figure S1) showed higher oscillation power. This participant (aged 7) was hypermetropic and his positive diopter lenses probably enlarged the stimuli resulting in stronger stimulation of the pupil. Elimination of this outlier did not alter the results.

Fixation dot and counting task conditions did not differ in pupil response amplitudes. However, all children seemed to enjoy the counting task the most. Although pupil perimetry is an objective testing method, higher intrinsic motivation and attention seem to result in stronger pupil responses (5; 4; 16; 21; 29). Attention was drawn away only a couple times to the appearing Pikachu in the counting task, meaning that attention was still relatively often at the flickering stimuli, leading to strong pupil responses. On the contrary, attention was almost continuously drawn away from the flickering stimuli towards the central stimuli in the video condition, explaining the weaker pupil oscillations. Thus, providing an engaging and more enjoyable task during a diagnostic visual field test (e.g., a counting or object finding task) is a preferred method for young children. For this reason, some alternatives to SAP have already been introduced (18; 20; 27). The Behavioural Visual Field screening (BEFIE) test (13) and SVOP (20) are examples of visual field tests, specifically developed with very young and neurologically impaired children in mind, that are tolerated better than conventional SAP methods. To illustrate, the BEFIE test managed to shorten time-to-diagnosis of visual field defects substantially in children suffering from brain disease (28) whereas SAP methods are generally performed unreliably in young children due to inability to cooperate, lack of comprehension, and psycho-motor impairment (24; 25; 26; 37). Despite these efforts with subjective and/or confrontational and behavioral perimetry tests, pupil perimetry in VR may even enhance the reliability as well. Additionally, this study showed VRgcFPP is applicable in children as young as three years old, filling a clinical gap where reliable visual field testing up until now was extremely difficult.

Our novel virtual reality implementation of pupil perimetry successfully evoked pupil responses comparable to responses found in previous studies with adults (23; 29). Gaze-contingent flicker pupil perimetry, as well as other variations of pupil perimetry (11; 32), proved to objectively measure visual field defects. Our results support the application of a virtual reality version of pupil perimetry in children both in a busy clinical setting, and in a telemedicine setting, or even at familiar places for the child, such as home or school. The experimenters experienced no difficulties when conducting the experiment at the participants’ residence. Indeed, various VR-based perimetry methods using inexpensive or smartphone-based VR HMDs have recently been studied with telemedicine in mind (1; 6; 35, 36). Some feature subjective active report tasks comparable to SAP (17; 30; 36) and others apply eye tracking to objectively measure looking responses (8; 42) in order to assess the visual field. None, however, harnessed the objective pupillary responses to light stimuli like in pupil perimetry.

Gaze distance from fixation target was studied to investigate whether any of the fixation targets captured the child’s gaze best. Some of the older children (aged approximately 8 years or older) were more capable of inhibiting saccades during fixation. Children under the age of 6 experienced more trouble maintaining fixation; they seemed to lose interest in the fixation dot earlier than the older children. However, this conclusion is merely based on the qualitative inspection of the data and the sample size was too small to statistically differentiate between age groups.

A limitation to the current study comprises of the lack of assessment of diagnostic accuracy of the VRgcFPP method with respect to detecting scotomas as all children tested did not suffer from visual field defects. Next to that, the eye tracker used in the current HMD is of inferior quality when compared to eye trackers used in standard pupil perimetry (e.g., Eyelink 1000 or Tobii Pro Spectrum; 33). It is unclear whether the lower quality has impact on the intended use. We did find clear changes in pupil diameter in response to the flickering stimuli (Figure 2B), suggesting that, in line with previous work on gaze-contingent flicker pupil perimetry (23; 29), the apparatus offers the opportunity to measure differences in sensitivities across the visual field in patients and healthy observers; future experiments with pediatric and adult patients suffering from visual field loss and comparative studies between more expensive eye tracking systems and the VR system used in this study might help shed some light on questions about diagnostic accuracy and applicability of eye-tracking in VR. Since the VR apparatus is an off-the-shelf device, it could not be modified to the smaller head sizes of young children. This resulted in suboptimal calibration and relatively smaller pupil powers in our youngest participants (see S1, S15, and S18 in the Supplementary Figure S1).

To conclude, our results support the application of this virtual reality version of pupil perimetry (VRgcFPP) for binocularly testing the visual field of children in a busy clinical setting. The VR set-up appears to be an ideal apparatus for children to allow free range of movement, an engaging visual task, and reliable eye measurements. A fixation counting task is recommended for use of pupil perimetry in young children as they enjoyed it the most and it achieved pupil responses as strong as the generally used fixation dot.

### Ethics and Conflict of Interest

The authors declare that the contents of the article are in agreement with the ethics described in http://biblio.unibe.ch/portale/elibrary/BOP/jemr/ethics.html and that there is no conflict of interest regarding the publication of this paper.

### Acknowledgements

This work was supported by a grant from UitZicht (grant 2017-18, funds involved: the ODAS foundation [grant number 2017-03]; the Rotterdamse Stichting Blindenbelangen [grant number B20170004]; and the F.P. Fischer Foundation [grant number 170511]), and a grant from the Janivo Foundation [grant number 2017170]. M.N. is supported by a grant from UitZicht (grant 2018-10, fund involved: Rotterdamse Stichting Blindenbelangen).

## supplementary material


