# Supplementary material for: Maintaining fixation by children in a virtual reality version of pupil perimetry
Source: J Eye Mov Res. 2022 Sep 19;15(3):10.16910/jemr.15.3.2. doi: 10.16910/jemr.15.3.2 (PMC10115433; doi:10.16910/jemr.15.3.2)
Supplement: Supplementary file 1 [file jemr-15-03-b-SD1-01.pdf]

Supplementary materials

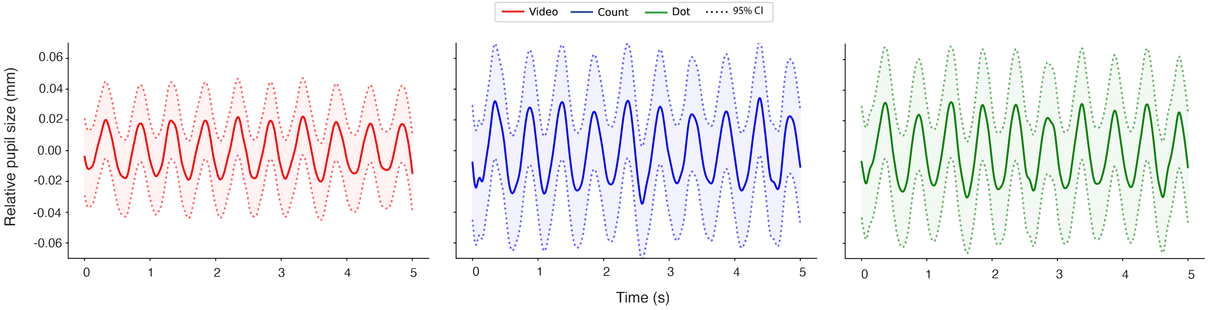

Figure S1. Relative pupil diameter over time with 95% confidence intervals for all subjects across fixation target conditions are shown in red (left panel) for the video fixation target, blue (middle panel) for the counting task fixation target, and green (right panel) for the fixation dot target in green. Pupil traces are averaged across stimulus locations and participants.

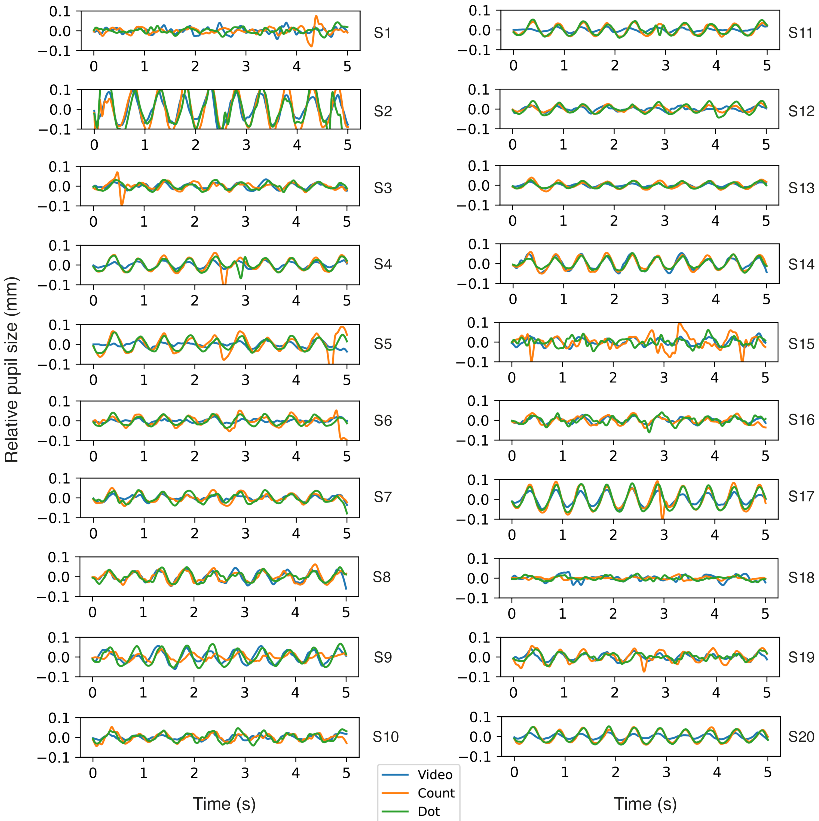

Figure S2. Average pupil traces (i.e., relative change in pupil diameter from baseline) for all subjects (S1-S20) per fixation condition (colors) over time. Pupil traces are averaged across stimulus locations.

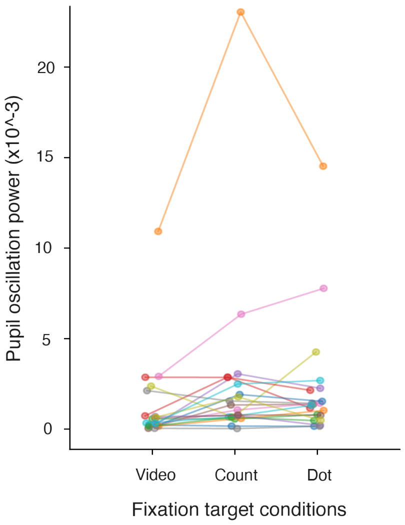

Figure S3. Average pupil amplitudes across fixation target conditions per participant (colors)

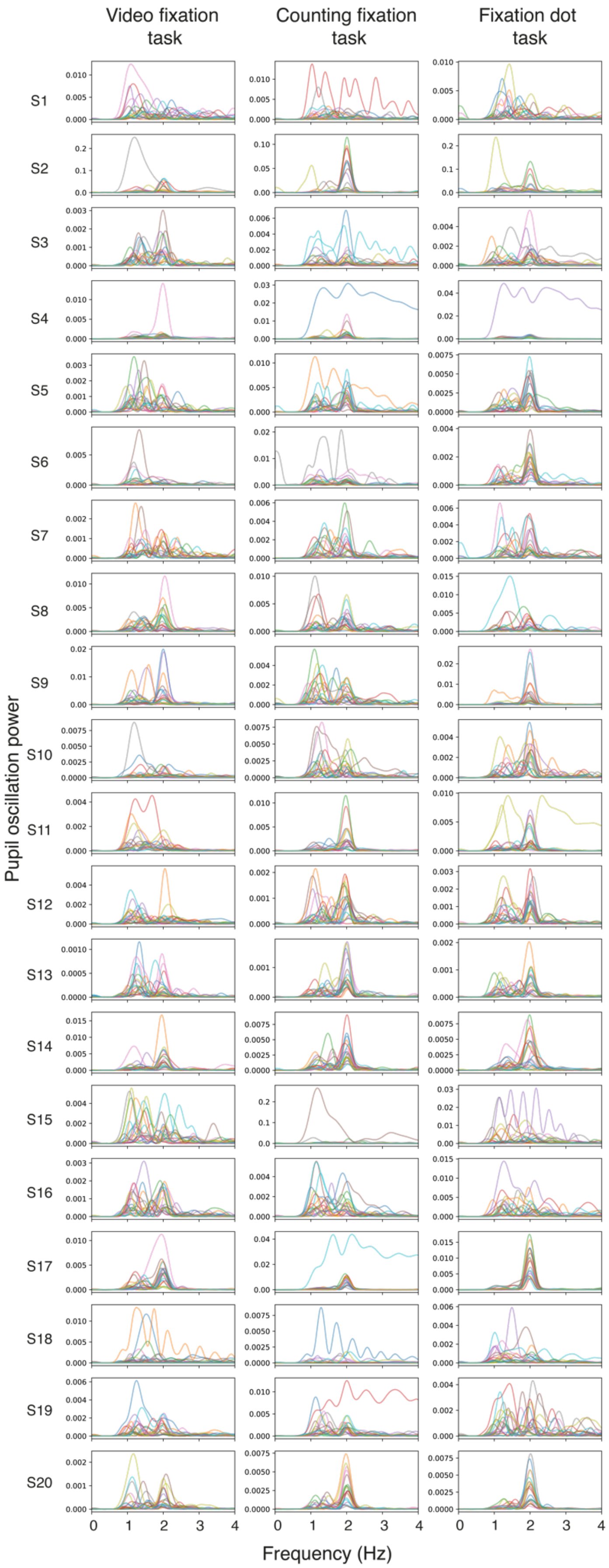

Figure S4. Pupil oscillation power spectra per trial (i.e., stimulus location; different colors) across participants and fixation conditions.
